# Physicochemical Profile, Antioxidant and Antimicrobial Activities of Honeys Produced in Minas Gerais (Brazil)

**DOI:** 10.3390/antibiotics11101429

**Published:** 2022-10-18

**Authors:** Vanessa de A. Royo, Dario A. de Oliveira, Pedro Henrique F. Veloso, Verônica de M. Sacramento, Ellen L. A. Olimpio, Luciano F. de Souza, Nathália da C. Pires, Carlos Henrique G. Martins, Mariana B. Santiago, Tânia Maria de A. Alves, Thaís M. Acácio, Afrânio F. de Melo Junior, Murilo M. Brandão, Elytania V. Menezes

**Affiliations:** 1 Laboratory of Natural Products, Department of General Biology, Universidade Estadual de Montes Claros, Montes Claros 39401-089, MG, Brazil; 2 Laboratory of Bioprospecting and Genetic Resources, Department of General Biology, Universidade Estadual de Montes Claros, Montes Claros 39401-089, MG, Brazil; 3 Cooperative of Beekeepers and Family Farmers of Northern Minas, Fazenda Bahia s/n, Bocaiuva 39390-000, MG, Brazil; 4 Laboratory of Microbiology, Institute of Biomedical Sciences, Department of Microbiology, Federal University of Uberlândia, Uberlândia 38408-100, MG, Brazil; 5 Laboratory of Chemistry of Natural Bioactive Products, Instituto René Rachou—Fiocruz Minas, Belo Horizonte 30190-002, MG, Brazil

**Keywords:** bee’s honey, phenolic compounds, pollen

## Abstract

Honeys can be classified as polyfloral or monofloral and have been extensively studied due to an increased interest in their consumption. There is concern with the correct identification of their flowering, the use of analyses that guarantee their physicochemical quality and the quantification of some compounds such as phenolics, to determine their antioxidant and antimicrobial action. This study aims at botanical identification, physicochemical analyses, and the determination of total polyphenols, chromatographic profile and antiradical and antimicrobial activity of honey from different regions of Minas Gerais. Seven different samples were analyzed for the presence of pollen, and color determination. The physicochemical analyses performed were total acidity, moisture, HMF, reducing sugar, and apparent sucrose. The compound profile was determined by UHPLC/MS, the determination of total phenolics and antiradical activity (DPPH method) were performed by spectrophotometry, and minimum inhibitory and bacterial concentrations were determined for cariogenic bacteria. All honey samples met the quality standards required by international legislation, twenty compounds were detected as the main ones, the polyfloral honey was the only honey that inhibited all of the bacteria tested. Sample M6 (Coffee) was the one with the highest amount of total polyphenols, while the lowest was M4 (Cipó-uva). Regarding the antioxidant activity, M5 (Velame) had the best result and M4 (Cipó-uva) was the one that least inhibited oxidation. Of the polyfloral honeys, there was not as high a concentration of phenolic compounds as in the others. Coffee, Aroeira, Velame and Polyfloral have the best anti-radical actions. Betônica, Aroeira, Cipó-uva and Pequi inhibited only some bacteria. The best bacterial inhibition results are from Polyfloral.

## 1. Introduction

Honey is a viscous and aromatic product made by bees from the nectar of flowers or honeydew. A natural sweetener, it has a complex composition of carbohydrates and other substances such as organic acids, amino acids, proteins, minerals, vitamins, lipids, aromatic compounds, flavonoids, pigments, waxes, pollen grains, various enzymes and other phytochemicals [1,2]. The presence of phenolic compounds in a composition is related to its antioxidant capacity, in addition to the antibacterial activity and stimulating effect on the multiplication of probiotic bacteria [3,4,5,6,7].

The characteristics of honey vary according to the botanical source and geographical origin, as well as climatic, processing and storage conditions [8]. The grains of different species are grouped according to their relative frequencies, thus, they can be classified as monofloral, with dominant flowering, or polyfloral when they come from different floral origins and do not have dominant pollen [9,10].

Thus, the nectar collected by bees for the production of honey, infers differences in composition. Therefore, this variation allows one to obtain different properties, such as biological activities [11,12]. Antibacterial activity is one of the most reported biological properties, with many studies demonstrating that honey is active against clinically important pathogens [2,13]. The intrinsic characteristics and the complex composition of honey, in which different substances with antimicrobial properties are included, make it an antimicrobial agent with multiple and different target sites in the fight against bacteria. This aspect, together with the difficulty of developing resistance to honey, indicates that it could become an effective alternative in the treatment of antibiotic-resistant bacteria, against which honey has already been shown to be effective [14].

Honey is a widely consumed natural product, not only for its taste and nutritional value, but also for its health benefits [15]. Bioactive compounds such as phenolics are present in honey in smaller proportions, but are responsible for some biological properties, such as antioxidants, that improve cell protection, help to prevent diseases and help to control aging. Some compounds that have been identified in honeys are gallic acid, vanillic acid, morin, *p*-hydroxybenzoic acid, *p*-methoxycinnamic acid, among others [16,17,18,19].

Honeys have been used for a long time in folk medicine [20,21], both orally and topically against various diseases: antimicrobial, gastrointestinal with protective and antioxidant properties, in addition to being a good source of energy [22]. With the greater dissemination of studies, there has been a growing interest in honey’s medicinal use, as well as in their treatment of diseases caused by oxidative stress, and in their anti-inflammatory, antiviral, antifungal, antitumor properties [23,24,25].

Their antimicrobial properties are directly related to their geographical origin, which gives each type of honey its own characteristics [26]. The floristic diversity of the region and the time of year in which flowering occurs interfere with coloration. Darker honeys are related to calcium and iron contents, and lighter honeys are correlated with sodium contents. Darker honeys have had better results related to antimicrobial activity than light colored honeys [27].

Honeys reduce prostaglandin levels and raise nitric oxide end products. These properties may help explain some of the biological and therapeutic properties of honey, particularly as an antibacterial or healing agent, which include stimulation of tissue growth, enhanced epithelialization, and minimized scar formation. These effects are ascribed to honey’s acidity, hydrogen peroxide content, osmotic effect, nutritional and antioxidant contents, stimulation of immunity, and to unidentified compounds [28].

Due to the growing need for natural alternatives for the treatment of several diseases, especially those related to antioxidant and antimicrobial properties, there is a need for more studies on monofloral or predominant flowering honeys. Thus, the objective of this work is to perform physical-chemical analysis, to obtain a pollen count for flowering classification, to obtain the chromatographic profile and to verify the antioxidant and antimicrobial activities in honeys from different locations in Minas Gerais.

## 2. Results

### 2.1. Botanical Identification

The pollen count analysis was performed by dissolving 10 g of honey in 20 mL of distilled water, the sediment was included in unstained glycerin gelatin after centrifugation, the slides were then mounted and, finally, sealed with paraffin [29,30]. The slides were taken for observation under a microscope, where the pollen was observed, counted and classified using the reference laminar of pollen grains PROBEE Ltd. and the database of images of bee plants in the state of Minas Gerais.

Honeys with pollen above 90% were considered monofloral and those between 45% and 89% were considered to have predominant flowering. Of the seven honeys analyzed, three of them were monofloral, three were considered predominant flowering and only one was polyfloral, with the main pollens *Baccharis* and *Croton* (Table 1). In Table 2, besides the scientific name of the main pollen, the family and popular name of the species are described.

### 2.2. Determination of the Honey Color

The staining was determined and followed the classification of the Pfund table [31]. Of the seven honeys analyzed, two were extremely clear, or extra-light amber (Cipó-uva and Velame), one was considered dark amber (Aroeira), one amber (Pequi), and the others were classified as light amber (Table 3).

### 2.3. Physicochemical Analyses

#### Total Acidity

Regarding acidity, it is possible to observe that they are towards the limit of what can be considered adequate. The honey from Cipó-uva (16.00 meq kg^−1^) was the one with the lowest total acidity content, followed by Polyfloral (18.43 meq kg^−1^). Betônica was the honey with the highest acidity (32.01 meq kg^−1^). In the analysis carried out, it was possible to determine that all honeys are within the established limit of 50 meq kg^−1^ (Table 4).

### 2.4. Moisture

In the determination of moisture, it was observed that the honey with the lowest moisture value is that of Cipó-uva (17.5%), the others have results between 18.0% and 19.5%, but also all within the limit, where the maximum allowed is 20% (20 g 100 g^−1^ of sample) (Table 4).

#### 2.4.1. Hydroxymethylfurfural

The maximum allowed limit is 60 mg kg^−1^ and all the results calculated for the honeys are within the limit. It can be observed that the honey with the highest HMF content was Coffee (55.70 mg kg^−1^) and Pequi (55.17 mg kg^−1^), whereas the honeys with the lowest levels are Polyfloral (2.47 mg kg^−1^) and Aroeira (9.77 mg kg^−1^) (Table 4).

#### 2.4.2. Reducing Sugars and Apparent Sucrose

For the determination of reducing sugars after the titration, the percentages were calculated and the honey with the highest values were Velame and Polyfloral, both with 74.07% and the lowest value was Aroeira with 66.66%. The minimum limit for reducing sugars is 65% (65 g 100 g^−1^ of sample) and all analyzed honeys were within the recommended limit (Table 4).

The maximal admissible value of this test for apparent sucrose is 6% (6 g 100 g^−1^ of sample), all were within the limit. The honey with the lowest value was that of Aroeira (2.31%) and the one with the highest value was that of Betônica (5.49%) (Table 4).

### 2.5. Total Polyphenols

The performance of total polyphenols and antioxidant activity of the honeys were evaluated by the boxplot pattern (Figure S1) and expressed in Table 5.

Total polyphenols levels were determined by the Folin–Ciocalteu method, read at 760 nm, with R^2^ = 0.998 determined on the gallic acid standard curve. It can be observed that the highest values found for the analyzed honeys were for Coffee (84.77 ± 0.05 milligrams equivalent to gallic acid per hundred gram of honey mgGAE 100 g^−1^) and Aroeira (74.74 ± 0.12 mgGAE 100 g^−1^) followed by Velame (70.06 ± 0.03 mgGAE 100 g^−1^). The other results follow in the sequence of Betônica (57.62 ± 0.0.07 mgGAE 100 g^−1^), Pequi (54.37 ± 0.03 mgGAE 100 g^−1^), Polyfloral (52.37 ± 0.03 mgGAE 100 g^−1^). Cipó-uva honey was the one with the lowest content found for total polyphenols (40.70 ± 0.03 mgGAE 100 g^−1^) (Table 4).

### 2.6. LC/MS/MS Analysis

The extraction process using ethyl acetate afforded fractions (0.1% yield) that were chromatographed using UHPLC-MS/MS system described in the Material and Methods section. The compounds with relative peak area above 30% eluted between 4.0 and 8.0 min. Twenty compounds were detected as major compounds within the seven samples, while only three, eluted at 1.1 and 1.2 min, had their *m/z* detected at 179.0561; 195.0511 and 177.0406. According to SmartFormula from Bruker, these m/z are compatible with the ion formulae [M-H] C_6_H_11_O_6_, C_6_H_11_O_7_ and C_6_H_11_O_6_, respectively. The main compounds from each sample presented the following data: *m/z* 199.0976, [M-H] C_10_H_15_O_4_ eluted at 6.6 min and detected in the Betônica, Pequi and Cipó-uva samples; *m/z* 263.1290, [M-H] C_15_H_19_O_4_ eluted at 6.6 min and detected in the Polyfloral sample; and *m/z* 263.1293, [M-H] C_15_H_19_O_4_ eluted at 6.9 min displaying a fragmented ion with *m/z* 153.0921, [I-H] C_15_H_19_O_4_, detected in the Velame, Aroeira and Coffee samples. The data from all UHPLC-MS/MS are registered in Table 5. The chromatographic profiles can be seen in the Supplementary Material in Figures S2–S8.

In the other formulas with relative areas below 30%, we searched for bibliographic references and the data are shown in Table 6.

### 2.7. Antiradical Activity

In determining the antioxidant capacity, the test with 2,2-diphenyl-1-picrylhydrazyl (DPPH) was used. To determine EC_50_ in mg mL^−1^ (Table 7; Figure S1), the equations of the standard lines for gallic acid and for each of the honeys were used. The R^2^ of the equations were between 0.944 and 0.997. The samples of honey from Velame (51.48 ± 1.48 mg mL^−1^), Aroeira (68.81 ± 2.36 mg mL^−1^) and Polyfloral (72.84 ± 0.27 mg mL^−1^) were the ones with the best observed antioxidant capacity. They were followed, in decreasing order, by Betônica (76.21 ± 3.29 mg mL^−1^), Coffee (77.69 ± 3.55 mg mL^−1^), and Pequi (105.56 ± 2.94 mg mL^−1^). Finally, the lowest DPPH-free radical scavenging activity was reported by the honey from Cipo-uva (150.71 ± 2.56 mg mL^−1^).

### 2.8. Antibacterial Activity Assay

Polyfloral honey was able to inhibit the growth of all microorganisms tested and the only one with MIC and MBC results for *E. faecalis*, *S. mitis*, *L. paracasei*, and *S. mutans*. The MIC for *S. salivarius* was observed for Polyfloral, Pequi and Aroeira honeys, but only Polyfloral and Pequi had MBC determined. For *S. sanguinis* all honeys had determined MICs. The best results were Betônica, Aroeira and Polifloral and for MBC the best were Polifloral and Cipó-uva. As a positive control, Chlorhexidine was used at concentrations from 0.000012% to 0.0059% (Table 8).

## 3. Discussion

### 3.1. Botanical Identification

The honeys were submitted to qualitative or quantitative microscopic analysis. Here, observation of the pollen grain and the pollen spectrum indicate the plants visited by the bees, which allows characterization based on botanical origin [37].

Honeys can be called heterofloral or wild, coming from the nectar of several plant species and monofloral or unifloral, which are undoubtedly the most attractive. It is possible to determine a honey’s origin from flowers by recognizing the dominant pollen grains [28].

The pollen grains of the different species were grouped according to relative frequencies. Where the species represents more than 45% of the pollen grain it is considered dominant pollen and it is considered accessory pollen when it represents between 15% and 45% [30]. Thus, it can be observed that six of the samples have dominant pollen, one of them with 99% (*Caryocar brasiliensis*), two with values above 90% (*Astronium urundeuva*: 94.34% and *Coffea arabica*: 90.09%), two above 83% (*Serjania lethalis*: 83.33% and *Croton urucurana*: 83.34%), one with 69.38% (*Hyptis* sp.) and finally one of them can be considered Polyfloral with the presence of pollen from several species where two of these are accessory pollen (*Baccharis calvescens*: 25.42% and *Croton urucurana*: 16.95%) (Table 1).

Monofloral honeys which are produced mainly from the nectar of single plant species are known as high quality products and they have a higher market price [38].

### 3.2. Determination of the Honey Color

The color of a honey is also related to its antioxidant capacity. The antioxidant action varies directly proportional with the color, which may be linked to the presence of anthocyanin and flavone groups [39]. Other relationships are with the floral origin, climatic factors during the flow of nectar and the temperature at which the honey matures inside the hives [40].

When the evaluation of honey by the market is based only on appearance, most of the time the consumer in the world market chooses light-colored honeys, which fetch higher prices than dark-colored honeys [41]. However, with more studies having been carried out, results of interest have been observed in dark honeys. Dark honeys are richer in minerals, have higher concentrations of calcium, iron, vitamin B, and vitamin C, and have a stronger aroma compared to light honeys, which have a shown a higher sodium concentration [27,41].

In a study with polyfloral honeys from the Vale do Jequitinhonha/Brazil, the variation determined for the samples was between 0.4244 (light amber) to 1.6059 nm (dark amber) [42]. Similar color results were observed in a previous study carried out by our group, where for Aroeira honeys showed dark amber, Pequi and Betônica showed amber and Velame showed light amber. However, Cipó-uva showed as extra light amber in this study (Table 3) but dark amber in the previously published study. This difference can occur between honeys of the same species, as the color depends on several factors, in which case it is possible to observe a difference in the composition of pollen [6].

### 3.3. Physicochemical Analyses

Honey is a complex mixture of various substances, and its composition depends on both floral and geographical origins, as well as anthropogenic factors. The precise identification of the origin guarantees the satisfaction of consumers’ needs and has an impact on the market value [43].

#### 3.3.1. Total Acidity

Acidity between 42.96 to 107.4 meq kg^−1^ was determined for Polyfloral honeys from Ethiopia, where the lowest content was much higher than in this study [44]. The honey with the highest acidity content was Betônica (32.01 meq kg^−1^) (Table 4), but was still well below the recommended limit of 50 meq kg^−1^ [31]. The values were lower than those found for honeys studied in the Jequitinhonha Valley, Brazil (42 meq kg^−1^) [42], and for some honeys from Kosovo (23.90 and 69.00 meq kg^−1^) [45]. In another study with honey produced in the Marsabit Forest Reserve of northern Kenya, levels between 19.00 and 23.00 meq kg^−1^ were observed [46]. Honeys with higher acidity content and values within accepted limits, such as Betônica and Pequi, may be of interest for antimicrobial evaluation studies. Acidity occurs due to the natural process of fermentation of honey and is related to its antibacterial efficacy, this occurs due to the presence of certain organic acids and the action of the enzyme glucose oxidase that originates gluconic acid, an extremely potent antibacterial agent [47,48].

#### 3.3.2. Moisture

Moisture is considered an important quality parameter used to determine the degree of ripeness of honey [49], where honeys with high water content can be a result of premature harvesting [50]. Moisture affects honey density, flavor, color, crystallization and fermentation [12].

Honeys from *Apis mellifera* bees are considered for consumption when their moisture content is below 20% [31]. The honeys studied were close to the upper limit, but none exceeded 20%, and the results found in this study are between 17.5% and 19.5% (Table 4). The lowest contents were, respectively, for Cipó-uva and Coffee honeys, these values are slightly higher than those observed in Romania (between 15.25 and 17.31%) [51], but are close to those of Vojvodina in the Republic of Serbia (between 17.8 and 16.6%) [52].

When compared to honeys from Kosovo that have contents between 14.0 and 19.0% [45], only Betônica is superior, but when compared to those from Vale do Jequitinhonha in Brazil (between 15.60% and 19.67%) [42], which is also a region of Minas Gerais, the results are similar. 

This index is important because in bee honeys with low moisture content, fermentation is more difficult [52].

#### 3.3.3. Hydroxymethylfurfural

The quality of honey can undergo some changes with the duration and temperature of storage, which essentially leads to the loss of enzymatic activities and the formation of hydroxymethylfurfural (HMF), a cyclic aldehyde that is produced by the degradation of sugars [50].

Formation of 5-Hydroxymethylfurfural is the result of reducing sugars in the presence of acid with increasing temperature and storage time by the Maillard reaction [32,53]. However, several factors can influence the formation of HMF in honey: temperature and heating time, storage conditions, use of metallic containers, and the chemical properties of honey, which are related to the floral origin [54].

In the results, it is possible to observe that the Coffee honey (55.70 mg kg^−1^) was the one that presented the highest calculated HMF value, followed by Pequi honey (55.17 mg kg^−1^) and Velame (50.53 mg kg^−1^), all are lower than those found in Uganda, East Africa, (69.6 mg kg^−1^) [37]. 

The Polyfloral (2.47 mg kg^−1^) presented the lowest value, followed by Aroeira honey (9.77 mg kg^−1^), Betônica (10.55 mg kg^−1^) and Cipó-uva (14.82 mg kg^−1^), respectively (Table 4), except for Polyfloral, all with values higher than those of Tanzania (6.6 mg kg^−1^) [37] and similar to those of Bejaia in Algeria (11.04 to 34.53 mg kg^−1^) [50]. 

In a study in Kosovo, the content was between 7.41 and 166.43 mg kg^−1^ [45], where some values were much higher than those allowed and in this research (2.47 to 55.70 mg kg^−1^) all the honeys studied were within the standards of honey in a tropical climate [31], different from that found for other Brazilian states, where some exceeded 60 mg kg^−1^, the limit established by Brazilian legislation [10], and one exceeded the limit of 80 mg kg^−1^ determined by the Council of the EU [31,55].

Higher HMF concentration is indicative of poor storage conditions and/or excess heating of honey [56].

Studies have been carried out with HMF, where they observed properties such as anti-inflammatory [57], antioxidant [58], and anti-allergic [59], but it is important to emphasize whereas other studies report cytotoxicity, mutagenicity, and carcinogenicity [60]. Safe dose levels are not yet well established, and further studies are needed.

#### 3.3.4. Reducing Sugars and Apparent Sucrose

Honey is made up of 70–85% of sugars, among them monosaccharides (fructose and glucose) in higher concentration and disaccharides, trisaccharides and higher sugars to a lesser extent. Bees convert disaccharides and trisaccharides from nectar to monosaccharides (example: sucrose to glucose and fructose), maltose and maltotriose to glucose [12,61,62,63].

Adulteration leads to a significant increase in sucrose content as well as a decrease in reducing sugars [61] and is usually achieved by mixing with different cheap sugar syrups or indirectly feeding these sugars to bees [63,64].

Regarding the results observed for reducing sugars of minimum 65% (66.66% to 74.07%) and maximum apparent sucrose content of 6% (2.31 to 5.59%) (Table 4), all samples evaluated are within the allowed limits [31].

The levels observed in this study differ from others carried out with honey from southern Brazil, where the results for reducing sugars and apparent sucrose did not comply with the legislation [65] but are compatible with a study carried out in Cuiabá, Brazil (57.72% to 71.29%) [66], two studies from Ethiopia (67.5 to 70.2%) [67], (61.45 to 71.41%) [68] and Kenya (73.03%) [69].

For the apparent sucrose contents, the studied honeys showed no changes and were lower in that value than those observed in Cuiabá/Brazil (15.20% to 21.10%) [66] where the values are considered outside the accepted limit. The results corroborate the studies from Ethiopia (2.57% to 5.17%) [67], (2.96 to 4.73%) [68] and Kenya (2.43%) [12].

### 3.4. Total Polyphenols

Polyphenols are a heterogeneous class of chemical compounds that can be divided into flavonoids (flavonols, flavones, flavanols, flavanones, anthocyanidin, chalcones, and isoflavones) and non-flavonoids (phenolic acids) [69]. More than 200 polyphenolic compounds have been identified in various honey samples [70]. These are essential components of honey found in small amounts and generated from the pollen of plants frequently visited by bees [71]. These substances have been recognized as the main responsible for the antioxidant activity of honey, related to the ability to scavenge free radicals [72], in biological and functional activity [73], the anti-inflammatory, antimicrobial and anticancer capacity [74].

In this research, the content of phenolic compounds varied from 40.70 to 84.77 milligrams, equivalent to gallic acid per hundred gram of honey (mgGAE 100 g^−1^). In a study carried out with Polish honeys, there were observed variations from 41.80 to 128.00 mgGAE 100 g^−1^ [75] and in a study in Estonia, the levels were from 26.2 to 88.7 mgGAE 100 g^−1^ [76]. Honeys studied around the world that showed similar levels to those observed in this study include those studies in Portugal (72.78 mgGAE 100 g^−1^) [77], Cuba (59.58 mgGAE 100 g^−1^) [78] and Malaysia (59.05 mgGAE 100 g^−1^) [79]. 

The total phenol contents varied considerably among the various honey samples [80]. Comparing the results observed in this study with another previous study by our research group, it is observed that the values found for Aroeira honey (74.74 mgGAE 100 g^−1^) were very similar in one of the honeys (Aroeira-A5 = 72.02 mgGAE 100 g^−1^), greater than one (Aroeira-A7 = 54.91 mgGAE 100 g^−1^) and less than two others (Aroeira-A1 = 101.67 mgGAE 100 g^−1^ and A6 = 81.63 mgGAE 100 g^−1^) [6], also lower than that studied in Janaúba-MG (99.68 mgGAE 100 g^−1^) [81]. 

For Cipó-uva (40.70 mgGAE 100 g^−1^) a similar result was obtained (A12 = 42.52 ± mgGAE 100 g^−1^) in a sample from the same region [6]. For Betônica honey (57.62 mgGAE 100 g^−1^) the value was slightly lower than in the previous study (Betônica-A3 = 62.95 mgGAE 100 g^−1^) and that of Pequi (54.37 mgGAE 100 g^−1^) has slightly higher values (Pequi-A8 = 48.82 mgGAE 100 g^−1^), whereas for Velame honey (70.06 mgGAE 100 g^−1^) the result was much higher than previously published (Velame-A14 = 45.52 mgGAE 100 g^−1^) [6]. Even for honeys collected in the same place, in different years, variations in the results of the contents of compounds may occur.

The effect of honey on human health depends on the bioavailability of the phytochemical compounds, and on their methods of absorption and metabolization [74]. Regarding, for example, flavonoids in honey, some glycosidases derived from the salivary glands of bees can cause hydrolysis of these compounds and therefore they are found as phenolic aglycones, which are easily absorbed through intestinal barriers, increasing their bioavailability compared to the same glycosylated flavonoids present in different food matrices [82].

### 3.5. LC/MS/MS Analysis

Four of the analyzed honeys have hexose (C_6_H_12_0_6_) which may be related to the presence of fructose and glucose found naturally in honeys [83]. Only in Pequi were C_6_H_12_O_7_ and C_6_H_10_O_7_ observed. C_6_H_12_O_7_, possibly gluconic acid, is abundant in plants, fruits, honey and used in the formulation of food, pharmaceutical and hygiene products [84]. C_6_H_10_O_7_ may be related to glucono-delta-lactone. This occurs due to the presence of glucose that reacts with atmospheric O_2_ and oxidizes it to gluconic acid (present in up to 1% in honey), the amounts are greater due to the presence of the enzyme glucose oxidase, where oxidation of the aldehyde group on C-1 β -D-glucose to a carboxyl group causes the production of glucono-delta-lactone (C_6_H_10_O_6_) and H_2_O_2_ [85,86] (Table 5).

In Aroeira, Pequi and Cipo-uva, the formula C_9_H_10_O_4_ was observed, which has already been identified in honeys of *L. scoparium* from Tasmania, New Zealand and *K. ambigua* as 4-hydroxyphenyllactic acid. This compound is present in the classification of Manuka honey, where minimum concentrations of 1 mg.kg^−1^ are required [87] (Table 5).

The formula C_10_H_12_O_4_ can be observed in Aroeira and Cipó-uva with a higher percentage of relative area, but it is also present in Polyfloral, where it was reported as hydroxyconiferyl alcohol in a study with honeys from Iran. Furthermore, the formula C_9_H_16_O_4_ can correspond to azelaic acid and is present in all honeys, but in Cipo-uva with the largest relative area [32]. Azelaic acid has several pharmacological uses in dermatology. Its anti-inflammatory and antioxidant properties are thought to be correlated with its effectiveness in papulopustular rosacea and acne vulgaris, among other skin conditions [88] (Table 5).

C_10_H_16_O_4_ was detected in a study in Korea with honeys prepared from sugar cane and beets [89]. Iranian honeys were determined to include succinic acid monocyclohexyl ester [32], and in our study the formula was detected in all but Polyfloral.

The formula C_9_H_10_O_3_ is present in coffee honey and has been reported in samples of native honeys as L-(-)-phenylatic acid [90] (Table 5). 

Polyfloral honey is that with the highest relative area for C_15_H_20_O_4_, but it is present in all honeys, it has been identified as abscisic acid in *Calluna vulgaris* honey produced in Portugal [91], Australia, and New Zealand *Leptospermum* [92]. This acid is also used for floral authentication when it has high levels [93], or for dietary supplementation of hives in temperate regions [94] (Table 5).

Sebacic acid (C_10_H_18_O_4_) was determined in Polish honey and in our study the formula was observed for Betônica, Pequi and Cipó-uva [95]. In a study carried out in Rio de Janeiro/Brazil, sebacic acid presented its antimicrobial mechanism through the permeability of the plasma membrane, triggering the production of NO and the induction of apoptosis [96] (Table 5).

Discussions for each of the compounds found in honeys in the literature (Table 6) are described below.

Rhamnetin, is a flavonoid class compound with pharmacological properties, including antioxidant, anticancer, anti-inflammatory, antiviral and antibacterial activity [97]. It has a corresponding formula in Betônica, Velame and Polyfloral.

Kaempferide is a naturally occurring flavonoid that has been isolated from the roots of *Alpinia officinarum* [98]. Like other flavonoids, it has antioxidant properties, anticancer and antihypertensive effects [99,100] and studies have demonstrated that it helps preservation of cardiac function, reduction of oxidative stress, has anti-inflammatory effects, and aids in myocardial infarction size, and cardiomyocyte apoptosis in the case of a reperfusion injury schema [101]. It can be present in Cipó-uva, Coffee and Velame.

Hydroxypalmitic acid formula appears in Coffee, Velame and Polyfloral.

Many such phenolic and flavonoid compounds, including quercetin, kaempferol, apigenin, and caffeic acid, have antioxidant and anti-platelet potential, and hence may ameliorate cardiovascular diseases (CVDs) through various mechanisms, such as by decreasing oxidative stress and inhibiting blood platelet activation. Kaempferol is absent only in Aroeira, and apigenin only in Velame, while caffeic acid is present in all. Luteolin is a flavonoid that can act as an anticancer agent [102,103]. In neuroprotective effects in neuroinflammation and neurotrauma the luteolin can alleviate cognitive decline and enhance neuroprotection in neurodegenerative diseases and stroke [104]. Apoptosis-inducing antiproliferative activity has been exhibited by pinobanksin [105], but this formula was not observed in Aroeira and Cipó-uva [106].

Naringenin and dihydroxydecenoic acid were observed in all the samples, except for Aroeira and Coffee. Naringenin is a citrus flavonoid that has several biological activities such as sepsis, fulminant hepatitis, fibrosis and cancer [107]. Dihydroxydecenoic acid has an antineuroinflammatory effect and is the second most abundant fatty acid, but has been less studied in royal jelly, the results of these studies have revealed a new role for tumor suppressor p53 in the inhibition of neuroinflammation [108]. 

Galangin is a flavone, considered the bioactive constituent of galangal and honey, and seems to be present in Betônica, Coffee, Velame and Polyfloral. In general, galangin exhibits various pharmacological effects such as anti-inflammatory, antioxidant, anticancer and antiviral activities [109].

Apigenin is a flavonoid and its formula was observed only in Velame. Many studies have revealed that apigenin has cytostatic and cytotoxic effects on various cancer cells, prevents atherogenesis, hypertension, cardiac hypertrophy, ischemia/reperfusion-induced cardiac injury and autoimmune myocarditis, chemical-induced liver and ischemia/reperfusion injury, asthma, bleomycin-induced pulmonary fibrosis, abnormal behavior and oxygen deprivation, glucose/reperfusion-induced neural cell apoptosis, pancreatitis, type 2 diabetes and its complications, osteoporosis, and collagen-induced arthritis [110].

Pinocembrine is one of the most abundant flavonoids in propolis, it has remarkable pharmacological properties such as neuroprotection and anti-oxidation and is an anti-inflammatory. It has been approved by the China Food and Drug Administration (CFDA) as a new treatment drug for ischemic stroke and is currently undergoing phase II clinical trials [111]. It can be present in Velame and Polyfloral.

The flavone chrysin, which occurs naturally in many plants, honey, and propolis. exhibits many biological activities and pharmacological effects, including antioxidant, anti-inflammatory, anticancer, and antiviral properties [112]; however, we could not find the formula in Pequi or Coffee.

Royal jelly acids are of interest as royal jelly has anti-hypercholesterolemic activity, antimicrobial activity, anti-inflammatory activity, antitumor/antiproliferative activity, and neutrophilic/neuroprotective activity [113]. These properties were observed in Betônica, Pequi, Aroeira and Coffee.

Syringaldehyde, belonging to the family of phenolic aldehydes, is an important natural redox mediator of fermentation because it is mainly used as a food additive, as a flavor enhancer or flavoring agent [114].

Gallic acid was not observed in Polyfloral and quinolol was not observed in Coffee. Gallic acid, in addition to its antioxidant activity, has recently been studied in the treatment of cataract patients [115], and for its antitumor effects [116].

The *p*-coumaric acid (4-hydroxycinnamic acid) is a phenolic acid, its biological activities include antioxidant, anticancer, antimicrobial, antivirus, anti-inflammatory, antiplatelet, anxiolytic, antipyretic, analgesic and antiarthritis activities. Its mitigating effects against diabetes, obesity, and hyperlipemia [117] were observed in Pequi, Aroeira, Cipó-uva and Coffee.

In all of these, formulas compatible with vanillic acid were also observed. This is a derivative of benzoic acid which is used as a flavoring agent, preservative, and food additive in the food industry. It has various pharmacological properties such as antioxidant, anti-inflammatory, immuno-stimulating, neuroprotective, hepatoprotective, cardioprotective, and antiapoptotic [118]. The phenyllactic acid is an important broad-spectrum antimicrobial compound that inhibits the growth of undesirable microbes through multifaceted actions [119]. 

The *p*-hydroxybenzoic acid seems to be present in Cipó-uva, Coffee, Velame and Polyfloral, in one study it was tested for antifungal activity and when combined with 4-coumaric acid had an inhibitory effect on *C. gloeosporioides* in walnut fruits [120].

### 3.6. Antiradical Activity

Antioxidants are natural chemicals that are mostly found in plants. Their main role is as a defense mechanism that neutralizes free radicals and prevents harmful oxidative effects [121].

Oxidative stress is the basis of structural and functional damage to key biomolecules such as nucleic acids, lipids and proteins, these injuries lead to the development of many diseases [74], which produce cellular damage, which then leads to the manifestation of degenerative cardiovascular diseases, as well as cancer and aging [122,123]. Oxidative stress due to cellular metabolism and other physio-biochemical activities of the body demand the necessity of antioxidants in a diet, something which can be fulfilled by honey. Antioxidant and other biological properties of honey are greatly determined by the polyphenol composition [124].

Honey contains antioxidant compounds derived from pollen sources [125], these protect cells from free radical damage [126] and can inhibit oxidative reactions by increasing total cellular antioxidant capacity and eliminating reactive oxygen species, thus reducing DNA damage [127]. Recent studies indicate that the biological activity of honey can also be attributed to phenolic compounds and their antioxidant activity [128,129].

Phenolic compounds, due to their medicinal properties, make honey an attractive prophylactic entity for the prevention of chronic diseases associated with oxidative stress, including cancer, cardiovascular diseases, diabetes, respiratory diseases, hypertension, neurodegenerative diseases, etc. [130].

Interest in investigating the antioxidant potential of honeys and analyzing their phenolic and flavonoid compounds has increased [131]. Due to their medicinal and health effects when used as a natural food supplement, or as a conventional therapy, they may be a new antioxidant to reduce many of the diseases directly or indirectly associated with oxidative stress [132].

Despite promising reports of anti-inflammatory activity in vitro, well-designed clinical trials still need to be carried out to confirm the benefits of honeys from different botanical sources in diseases that include episodes of inflammation [123].

In honeys, the antioxidant capacity does not depend solely on phenolic compounds, but may be due to organic acids, amino acids, proteins, as well as Maillard reaction products [133].

The antioxidant properties of honey can be measured in the form of antiradical activity using the 1,1-diphenyl-2-picrylhydrazyl (DPPH) scavenging assay [129]. DPPH is a test method frequently used to analyze the antioxidant activity of honey, as it is very simple and fast and shows the overall antioxidant capacity of the sample, using solid and liquid samples [133].

In the observed results of EC_50_, Velame honey (51.48 ± 1.48 mg mL^−1^) was the one with the best antioxidant result. The lowest antioxidant activity was observed for honey from Cipó-uva (150.71 ± 2.56 mg mL^−1^), and even so, this was higher than that found in a study carried out in Piauí for honey from Cipó-uva (237.70 mg mL^−1^). Polyfloral honey (72.84 ± 0.27 mg mL^−1^) was also superior to the study from Piauí (136.92 mg mL^−1^) [129]. The average results are similar to a study carried out in Saudi Arabia with five wild honeys, the values of four of which were between 95.42 and 54.25 mg mL^−1^, with only one having a lower value (10.70 mg mL^−1^) [130]. When compared with the results of a previous study of our group, with honeys of the same flowering, it was possible to observe that most of the honeys were less effective (Table 5) [6].

In this evaluation, the Betônica, Aroeira, Coffee and Polyfloral honeys had a statistically similar performance with each other, where the EC_50_ ranged from 68.81 ± 2.36 mg mL^−1^ (M3) to 77.69 ± 3.55 mg mL^−1^ (M6). This last sample was similar to Betônica honey 76.21 ± 3.29 mg mL^−1^ (Table 7). It is important to note that the lower the EC_50_ value, the greater the efficiency of the sample in deactivating the free radical.

The antioxidant effect of honey is well established, but it is urgent to explore the exact mechanisms involved and extrapolate them to clinical trials. The exact antioxidant mechanism is unknown, but proposed mechanisms include free radical scavenging, hydrogen donation, metal ion chelation, hydroxyl flavonoid substrate action, and superoxide radical actions [134,135].

### 3.7. Antibacterial Activity

According to a World Health Organization oral health report published in 2022, minimally invasive intervention approaches to preventing and treating cavities should be applied to prolong the longevity of natural teeth and prevent unnecessary pain, infection, and permanent tooth damage [136].

In dentistry, honey has been used as a preventive or therapeutic remedy for some periodontal diseases mainly associated with bacteria, such as tooth decay. Findings demonstrate the pronounced antibacterial effect of different honeys against various periodontal pathogens, including *Streptococcus mutans* [137]. The acidogenic, sugar-fermenting species, *S. mutans*, is the main causative agent of dental caries; however, caries lesions are a consortium of microorganisms [138].

Polyfloral honey inhibited the growth of *S. mutans* with MIC and MBC at a concentration of 10%, a result superior to that found in a study carried out with honeys from Alexandria (Egypt), where four of the tested samples had MIC at a concentration of 50% [139], an Arabian honey inhibited bacterial growth at concentrations between 12.5% and 25% [140], while a natural Hamadan honey inhibited it at concentrations above 20% [141] and in another study there was no inhibition at concentrations of 5%, 10%, 20% and 40% [142].

Polyfloral honey was effective in terms of antimicrobial activity in relation to all strains tested and the only one capable of inhibiting it with MIC and MBC, showing results for *E. faecalis*, *S. mitis*, *L. paracasei*, *S. mutans*. In relation to *E. faecalis*, other honeys have already been tested and did not have satisfactory results, these include honey produced by *Appis dorsata*, where the concentrations used were above 25% [143], and Polyfloral honey with MIC and MBC in the concentration of 20%, even when the honey is from different bee species.

Regarding the determination of MIC and MBC of *S. mitis* and *L. paracasei* strains for Polyfloral honeys, inhibition results were obtained at concentrations of 20% and 10%, respectively, and results described in recent literature for comparison were not observed; however, in a study from 1994, the MIC of honey for *S. mutans*, *S. salivarius*, *S. sanguis* and *S. sobrinus* was 25%, higher than in this study [144].

The MIC of *S. salivarius* was determined for Polyfloral, Pequi and Aroeira honeys, but only Polyfloral and Pequi had MBC determined. In all honeys the MIC was determined for *S. sanguinis* with better inhibition observed for Betônica, Aroeira, Cipó-uva and Polyfloral. The best MBC was the Polyfloral, while Cipó-uva and Coffee was superior to 20% (Table 8).

There are several studies on the effectiveness of honey against Gram-positive bacteria, one of these reports that the MIC for *S. aureus* is 25% [145], in another study *S. aureus* and *P. aeruginosa* show MIC of 12.5% [146], values that corroborate with inhibition data determined in this study.

Efficacy concentrations of 30–40% were determined for MGO-400 Manuka honey and Gram-positive tested Polish honeys (*S. aureus*, *E. faecalis*, *E. faecium*) [147], honey from different regions of Serbia had activity against *S. aureus* (less than 50%) [148], while in ATCC strains in a study with Tilia honey all were resistant [149]. These reports are different from this study where some honeys inhibited bacteria from ATCC strains at concentrations of 10 to 20% (Table 6), results also observed for Acacia honey [149].

These different results emphasize how the diversity of monofloral honey, depending on the geographic area and climatic conditions for samples collected, or even the differences between the MIC values, can be justified by the different methods and techniques chosen to test the strains, or even by the variety of strains tested, such as Manuka honey [149].

In the case of the antimicrobial assay, the relationship between activity and dark color of the honeys was not observed [150] even though Pequi (amber) inhibited the growth of *S. salivarius* and Aroeira (dark amber) of *S. salivarius* and *S. sanguinis*, lighter honeys such as Betônica and Cipó-uva had results for *S. sanguinis* and the most promising was the Polyfloral that is light amber (Table 3), but our result corroborates the studies that showed that there was no relationship between color and antibacterial activity of honey, where some honeys of light color, such as orange blossom and clover, were more active as antibacterials against *Salmonella enteritidis* than darker honeys studied [151]. 

The inhibition mechanism may be related to the low pH of honey and the high sugar content that is sufficient to prevent the growth of microorganisms [152]. The association of high content of reducing sugars with antimicrobial activity is valid for Polyfloral honey, while for Betônica, Pequi and Aroeira it may be related to acidity. It is also worth noting that the honeys with the lowest HMF levels, mainly the Polyfloral, were those that had some reported antimicrobial activity, this parameter differs for the Pequi honey, which had an antimicrobial action with a high HMF index (Table 4).

Currently, a variety of natural products or their active ingredients, such as curcumin, honey, green tea extract and aloe vera, have become part of dental treatment due to their reduced toxicity, wide availability, and cost-effectiveness [153]. The antibacterial activity of honey can be enhanced by the presence of polyphenols, including flavonoids that are present in certain types of honey [154,155].

Polyphenols represent a group of biologically active secondary metabolites commonly found in honey, the polyphenolic composition is very diverse depending on botanical and geographic origins [156]. Thus, polyphenols, frequently reported in honey samples, can contribute to antibacterial activity, acting directly by producing H_2_O_2_ and reducing Fe(III) to Fe(II), which triggers the Fenton reaction to create more potent reactive oxygen species, such as hydroxyl radicals. A key factor in determining whether polyphenolic compounds exhibit antioxidant or antibacterial properties is the pH value [157].

Progress in clinical application opens new avenues for antimicrobial use in the treatment of periodontal diseases [158]. Honey is often used as a mouthwash in clinical trials to treat periodontal disease [159]. Its anti-inflammatory activity, combined with its significant antioxidant content, may also be beneficial in preventing the erosion of periodontal tissues that occurs as collateral damage from free radicals released in the inflammatory response to infection [160,161].

## 4. Materials and Methods

### 4.1. Chemicals and Instruments

All reagents and chemicals used were analytical grade from Sigma Chemical Company (St. Louis, MO, USA). A spectrophotometer (Shimadzu UV-VIS 2550/Tokyo, Japan) was used for absorbance measurements and a refractometer (VODEX VX090/Vitória/ES, Brazil) for moisture determination.

### 4.2. Honey Samples

The seven bee honeys (*Apis mellifera*) analyzed were provided by COOPEMAPI, based in Bocaiuva-MG), samples were received by the cooperative in (January to June) 2022. The samples were identified by numbering and stored protected from light (25 to 30 °C). All methods are based on specialized literature, including the Codex Alimentarius, Association of Official Analytical Chemists (AOAC), Normative Instruction number 11 of 10/20/2000 [10,31,162] and publications of the International Honey Commission. Experiments were performed in triplicate and all results are shown as mean +/− SD.

### 4.3. Botanical Identification

The microscopic slides were prepared by dissolving 10 g of honey in 20 mL of deionized water. After centrifugation, the pellet was embedded in unstained glycerin gelatin and the slides sealed with paraffin. The amount of pollen of the species was observed and the result was interpreted by the dominance of pollen thiop. The pollen count analysis was performed as described by Barth, 2004 and Louveuax, et al., 1978 [29,30] and the reference sheet was PROBEE Ltd.

#### 4.3.1. Determination of the Honey Color

This analysis was performed according to the methodology proposed by the Codex Alimentarius Commission [31], which consists of reading the absorbance of the pure sample in a spectrophotometer at 560 nm against pure glycerin blank. Classification was performed according to the Pfund table.

#### 4.3.2. Determination of Acidity

The total acidity of honeys was obtained through the determination of free and lactonic acidity and was determined according to method No. 962.19 of AOAC (1998) [162], in which the sample was titrated for free acidity with a solution of NaOH 0.05 mol L^−1^, until it reached a pH of 8.5. For lactonic acidity, after the solution reached a pH of 8.5, 10 mL of 0.05 mol L^−1^ NaOH were pipetted and, with 0.05 mol L^−1^ HCl, the back titration was performed until pH 8.3.

#### 4.3.3. Determination of Moisture

The moisture was determined by refractometry, according to method no. 969.38 b of AOAC (1998) [162]. The principle of this method is based on the determination of the refractive index of honey at 20 °C, and for each degree above the temperature that the sample presented, 0.00023 was added. The corrected refractive index was converted to moisture percentage using a reference table.

#### 4.3.4. Determination of Hydroxymethylfurfural

The hydroxymethylfurfural (HMF) content was determined using the spectrophotometric method at 284 and 336 nm, according to method no. 980.23 of AOAC (1998) [162]. 5 g of bee honey was weighed in a beaker and transferred to a 50 mL flask. Then 25 mL of water was added, 0.5 mL of Carrez I solution and 0.5 mL of Carrez II solution, the resulting solution was homogenized and made up to 50 mL with deionized water. It was filtered through a quantitative filter paper, discarding the first 10 mL of the filtrate. Four test tubes were used to determine HMF. In the first tube, 5 mL of filtered solution and 5 mL of 0.2% sodium bisulfite solution were added, this tube being considered as a reference. In the others, 5 mL of the filtrate and 5 mL of deionized water were added, these are the test solutions. The test solutions were homogenized and measured in a UV-visible spectrophotometer at wavelengths 284 and 336 nm. Prior to the reading that was taken, the device was calibrated with the corresponding reference solution.

HMF mg 100 g^−1^ honey = (A284 − A336) × 14.97 × 5 g^−1^ of sample

#### 4.3.5. Determination of Reducing Sugars

The determination of reducing sugars was carried out according to the CAC method [163] from the modification of the Lane and Eynon procedure, involving the reduction of the Fehling solution, modified by Soxhlet, during the titration at boiling point with a solution of bee honey sugar reducers, using methylene blue as an indicator. The apparent sucrose content was determined after inversion by acid hydrolysis, according to the CAC method [163].

#### 4.3.6. Apparent Sucrose

An amount of 50 mL of the honey solution obtained in the determination of reducing sugars was pipetted into a 100 mL volumetric flask and 25 mL of water was added. Heating was carried out at 65 °C in a water bath. The flask was removed from the bath and 10 mL of hydrochloric acid solution was added and the solution was allowed to cool naturally to room temperature, then neutralized with sodium hydroxide solution.

P = sample mass in g
V1 = number of mL of diluted sample solution spent in the titration
C = number of g of invert sugar percent, obtained before inversion, reducing sugars [164].

### 4.4. Total Polyphenols

For the determination of the total polyphenols content in the investigated honey samples, we used the Folin–Ciocalteu method, which is a colorimetric in vitro assay measuring the total reducing capacity of a sample [165]. An accurately weighed 1 g sample of each honey was put in a 10 mL volumetric flask, which was completed with water and filtered through with paper weight 80 g/m^2^. An amount of 0.5 mL of this solution was then added with 2.5 mL Folin–Ciocalteu reagent (0.2 n), and mixed for 8 min followed by the addition of 2 mL of sodium carbonate (75 g L^−1^). Then the mixture solution was allowed to incubate at room temperature for 2 h and the absorbance was measured at 760 nm, while methanol was used as blank. All measurements were taken in triplicate, and then the results were averaged and plotted on a graph of (concentration/absorbance) to determine the equation of the line and R^2^. Gallic acid (3,3,4-trihydroxybenzoic acid in concentrations between 30–80 µg/mL) was used as a standard to derive the calibration curve. The total phenolic content was expressed in mg equivalent of gallic acid per 100 g of honey [165].

### 4.5. Preparation of Honey Extracts

For the preparation of the extracts an aqueous solution of methanol 50% (*v*/*v*) was used. Then, the bee honey was diluted (8 mL of honey bee to 80 mL of 50% methanol solution). This solution was kept in a reflux device for two hours at 80 °C. Dilution and extraction were performed on each bee honey sample separately, stored in sealed jars and kept in the freezer [166].

### 4.6. LC/MS/MS Analysis

The LC/MS/MS analysis was adapted from Keckes et al. [33].

#### 4.6.1. Extraction

Samples were transferred to pre-tared scintillation vials. Due to the viscosity of the samples, it was necessary to heat them in a water bath at 45 °C. An amount of 50 mL of NaCl solution (2% *w*/*v*) was prepared. In this way, 1 g of NaCl was dissolved in 50 mL of ultra-pure water. An amount of 5 mL of this solution was added to each sample. This material was vortexed for 10 s. Then extractions were performed with ethyl acetate (3x with 5 mL). The organic phase was separated and Na_2_SO_4_ was added thereto, stirred, filtered through cotton into a pre-tared scintillation vial. The solutions were dried in speedvac (Table 9).

#### 4.6.2. Preparation of Solutions for Analysis

A solution of 9 mL methanol + 6 mL H_2_O was prepared and the samples dissolved as indicated in Table 9. They were then taken to ultrasound for 10 min, centrifuged for 15 min, left at room temperature for about 24 h and finally 50 µL were transferred to automatic injection vials in the UHPLC. An amount of 1000 µL of methanol/water was added to the extraction control blank sample (without honey). The preparation of standards is described in Table 10.

#### 4.6.3. Analysis Method LC-MS/MS

The chromatograms were obtained using a UHPLC system consisting of a chromatograph with a Nexera LC-30AD pump, connected to an autosampler Nexera SIL-30AC and a DAD Nexera SPD-M20A detector, supervised by a CBM Nexera 20 A (Shimadzu, Japan). The chromatograph was coupled to a mass spectrometer with time-of-flight detection model maXis-ETD ESI-QqTOF (Bruker, Germany). Separations were performed on a Shimpack XR-ODSIII, C18, 2.2 um, 80 A, 2.0 × 150 mm column (Shimadzu, Japan). The mobile phase consisted of (A) water with 0.1% formic acid and (B) acetonitrile with 0.1% formic acid. A linear gradient from 5 to 95% B in 15 min was used. Between each injection of 5 µL of each sample, the column was reconditioned with 95% B for 3 min and 5% B for 6 min. The spectrometer operated as follows: Ion source type: ESI; negative polarity; scan 100 at 1500 *m/z*; nebulizer gas: 3.0 bar; drying gas flow: 8 L/min; temperature: 200 °C.

### 4.7. Antiradical Activity

The 2,2-diphenyl-1-picrylhydrazyl (DPPH) free radical scavenging activity of bee honey samples was determined as described by Brand-Williams et al. (1995) [167]. To carry out the test, the extract, at concentrations between 50 and 100%, was used (500 µL). A stock solution of DPPH 40 µL mL^−1^ of methanol was prepared, from which 3000 µL were taken and added to the sample, then shaken vigorously and kept in the dark for 25 min at 25 °C. To obtain the standard curve of gallic acid, a stock solution at 80 µg mL^−1^ was prepared and concentrations between 30 and 80 µg mL^−1^ were used the absorbance of the solution was measured at 517 nm, using a spectrophotometer (SHIMADZU—UV-VIS 2550/Tokyo, Japan) against a methanol blank. All measurements were taken in triplicate. With the absorbance values, the percentage of antioxidant activity was calculated by the equation:

{(AbsCont—AbsAmos)/AbsCont} × 100 [168], where:
AbsCont represents the absorbance value of the control;
AbsAmos represents the absorbance value of the sample.

### 4.8. Antibacterial Activity Assay

#### 4.8.1. Cariogenic Bacteria

The bacteria used in this study were obtained from the American Type Culture Collection (ATCC): *Streptococcus salivarius* (ATCC 25975), *S. mitis* (ATCC 49456), *S. sanguinis* (ATCC 10556), *S. mutans* (ATCC 25175), *S. sobrinus* (ATCC 33478), *Lactobacillus paracasei* (ATCC 11578), *Enterococcus faecalis* (ATCC 4082). All the bacteria were kept in the Laboratory of Antimicrobial Assays (LEA) of the Federal University of Uberlândia, Brazil at −20 °C, in 80% glycerol solution.

#### 4.8.2. Minimum Inhibitory Concentration and Minimum Bactericidal Concentration

Minimum inhibitory concentration (MIC) is defined as the lowest concentration of an extract, fraction or compound that can inhibit bacterial growth. The experiment was performed in 96-well microplates and repeated three times.

For the assays, stock solutions were prepared prior to each batch of testing at concentrations up to 20% (*w*/*v*) honey in Brain Heart Infusion broth (Difco, Detroit, MI, USA). Solutions were vortexed until completely dissolved, then sterilized by serial filtration through 0.22 μm polyethersulfone (PES) membranes (Millipore, Tullagreen, Carrigtwohill, Country Cork. Ireland) to eliminate contaminating spore-forming organisms The tested concentrations of the samples ranged from (% *w*/*v*) 0.009%, 0.019%, 0.039%, 0.078%, 0.15%, 0.3%, 0.6%, 1.25%, 2.5%, 5%, 10% and 20%, the control (chlorhexidine) was tested at concentrations between 0.000012% to 0.0059%, and the inoculi were adjusted to a cell concentration of 5 × 10^5^ CFU mL^−1^ [169]. Inoculated wells containing bacteria were only included to control growth. Noninoculated wells (without any bacteria) were also employed to ensure broth sterility. The 96-well microplates were incubated at 37 °C for 24 h. After incubation, 30 μL of 0.02% aqueous resazurin solution was added to each well to observe microbial growth. The blue and red colors represent the absence and presence of microbial growth, respectively [170].

MBC is defined as the lowest concentration of the sample where no bacterial growth occurs. A substance is considered to exert a bacteriostatic effect when its MBC value is higher than its MIC value. However, a substance is considered to exhibit a bactericidal effect when its MBC value is the same as its MIC value. To determine MBC, 10 μL of the inoculum, removed from each well before resazurin was added, was plated on blood agar supplemented with 5% defibrinated horse blood. The plates were incubated in a bacteriological oven or anaerobiosis chamber at 37 °C for 24 h [171].

### 4.9. Statistical Analysis

The results were performed in triplicate and expressed as mean ± standard deviation. We used the software R (4.1.0) for the statistical analyses. The data set were initially submitted to the Shapiro–Wilk normality test. Then, we used analysis of variance (ANOVA) for parametric data, with a posteriori Tukey test for comparisons between means, alpha level of 0.05. We built boxplot to evaluate the behavior of different honeys for total phenolics and antioxidant activity. The boxplot graphically represents the median, first and third quartiles. Like the standard deviation, the width of the box can be used to assess the dispersion of the data.

## 5. Conclusions

In our study, the honeys with the best results in terms of anti-radical action were Velame and Aroeira, followed by Coffee. These were also the honeys with the highest levels of total phenolics. Differences in phenolic compounds may be related to differences in geographic origin and floral sources. No relationship was found between color and the presence of phenolic compounds and antioxidant action. The Polyfloral honey was the only one that inhibited all the bacteria tested, followed by Aroeira, Pequi and Cipó-uva, honey and showed to be the best bactericidal. The chromatographic profile revealed compounds of interest that should be further studied. However, the presence of polyphenols offers a great perspective in dentistry; however, the relative effectiveness of monofloral and polyfloral honeys to be used in treatments must remain under investigation to better define their potential as a natural antioxidant or antimicrobial medicinal agent that can be administered alone or as an adjunct to therapy.

## Figures and Tables

**Table 1:** Pollen analysis in honeys.

	Pollen Type	Pollen Count	Index %
M1	*Hyptis* sp.	213	69.38
	*Croton urucurana*	30	9.77
	*Eucalyptus robusta*	28	9.12
	*Baccharis calvescens*	11	3.58
	*Astronium urundeuva*	10	3.26
	*Mimosa scabrella*	4	1.30
	*Protium* sp.	3	0.97
	*Sida* sp.	2	0.65
	*Serjania lethalis*	2	0.65
	*Cecropia glazioui*	2	0.65
	*Anadenanthera colubrina*	2	0.65
M2	*Caryocar brasilense*	200	99.00
	*Piptadenia communis*	1	0.50
	*Eucalyptus robusta*	1	0.50
M3	*Astronium urundeuva*	500	94.34
	*Eucalyptus robusta*	30	5.66
M4	*Serjania lethalis*	150	83.33
	*Astronium urundeuva*	30	16.66
M5	*Croton urucurana*	150	83.34
	*Eucalyptus robusta*	20	11.11
	*Anadenanthera colubrina*	10	5.55
M6	*Coffea arábica*	100	90.09
	*Baccharis calvescens*	5	4.50
	*Serjania lethalis*	2	1.80
	*Citrus sinensis*	2	1.80
	*Eucalyptus robusta*	1	0.90
	*Vernonia scorpioides*	1	0.90
M7	*Baccharis calvescens*	30	25.42
	*Hyptis* sp.	10	8.47
	*Myracrodum urundeuva*	15	12.71
	*Croton urucurana*	20	16.95
	*Ipomoea* sp.	16	13.56
	*Richardia* sp.	17	14.41
	*Serjania* sp.	5	4.24
	*Mimosa caesalpiniaefolia*	5	4.24

M1 = Betônica; M2 = Pequi; M3 = Aroeira; M4 = Cipó-uva; M5 = Velame; M6 = Coffee; M7 = Polyfloral.

**Table 2:** Identification of honeys of the according to predominant flowering.

Popular Name	Scientific Name	Botanical Family
Betônica	*Hyptis* sp.	Lamiaceae
Pequi	*Caryocar brasiliense* Cambess.	Caryocaraceae
Aroeira	*Astronium urundeuva* (M. Allemão) Engl.	Anacardeaceae
Cipó-uva	*Serjania lethalis* A. St.-Hil.	Sapindaceae
Velame	*Croton urucurana* Baill.	Euphorbiaceae
Coffee	*Coffea arabica* L.	Rubiaceae
Polyfloral	Varied flowering species	-

**Table 3:** Color shades of honeys (mean ± SD; *n* = 3).

Identification	Color	Result (nm)
Betônica	Light amber	0.211 ± 0.001 ^e^
Pequi	Amber	0.480 ± 0.001 ^b^
Aroeira	Dark amber	0.956 ± 0.001 ^a^
Cipó-uva	Extra light amber	0.186 ± 0.001 ^f^
Velame	Extra light amber	0.181 ± 0.001 ^g^
Coffee	Light amber	0.296 ± 0.001 ^c^
Polyfloral	Light amber	0.286 ± 0.001 ^d^

Means followed by the same letter in the column do not differ according to Tukey’s test at *p* *<* 0.001.

**Table 4:** Results of analysis and parameter limits for honeys (mean ± SD; *n* = 3).

	Acidity (meq kg^−1^)	Moisture (%)	HMF (mg kg^−1^)	Reducing Sugars (%)	Apparent Sucrose (%)
M1	32.01 ± 0.006 ^a^	19.5 ± 0.010 ^a^	10.55 ± 0.006 ^e^	71.43 ± 0.012 ^b^	5.49 ± 0.012 ^a^
M2	30.55 ± 0.010 ^b^	19.0 ± 0.012 ^b^	55.17 ± 0.010 ^b^	68.96 ± 0.012 ^d^	5.11 ± 0.012 ^b^
M3	28.13 ± 0.010 ^c^	18.5 ± 0.006 ^c^	9.77 ± 0.010 ^f^	66.66 ± 0.021 ^f^	2.31 ± 0.015 ^g^
M4	16.00 ± 0.015 ^g^	17.5 ± 0.012 ^e^	14.82 ± 0.012 ^d^	67.56 ± 0.015 ^e^	3.36 ± 0.015 ^d^
M5	22.31 ± 0.015 ^e^	19.0 ± 0.006 ^b^	50.53 ± 0.017 ^c^	74.07 ± 0.015 ^a^	2.91 ± 0.012 ^e^
M6	25.22 ± 0.012 ^d^	18.0 ± 0.021 ^d^	55.70 ± 0.015 ^a^	70.42 ± 0.010 ^c^	3.65 ± 0.015 ^c^
M7	18.43 ± 0.006 ^f^	19.0 ± 0.006 ^b^	2.47 ± 0.006 ^g^	74.07 ± 0.012 ^a^	2.85 ± 0.012 ^f^
Limits	Maximum 50 meq kg^−1^	20 g 100 g^−1^ (20%)	Maximum 60 mg kg^−1^	Minimum 65 g 100 g^−1^ (65%)	Maximum 6 g 100 g^−1^ (6%)

M1 = Betônica; M2 = Pequi; M3 = Aroeira; M4 = Cipó-uva; M5 = Velame; M6 = Coffee; M7 = Polyfloral. Means followed by the same letter in the line do not differ according to Tukey’s test at *p* < 0.001.

**Table 5:** Data from all samples for analyses of UHPLC/MS/MS.

RT (min) ^a^	RA (%) ^a^	*m/z* Íon Molecular [M-H]^-^^b^	MS/MS ^b^	Molecular Ion Formula ^c^	Molecular Formula ^c^	
1.1	49.64	179.0561		C_6_H_11_O_6_	C_6_H_12_O_6_	Pequi
	31.20					Betônica
	37.09					Cipó-uva
	32.70					Coffee
1.1	32.70	195.0511		C_6_H_11_O_7_	C_6_H_12_O_7_	Pequi
1.2	31.13	177.0406		C_6_H_9_O_6_	C_6_H_10_O_6_	Pequi
5.1	94.14	181.0506		C_9_H_9_O_4_	C_9_H_10_O_4_	Aroeira
	88.84					Cipó-uva
	37.62					Pequi
5.6	40.24	361.1509	199.0976	C_16_H_25_O_9_	C_16_H_26_O_9_	Pequi
	36.55			C_10_H_15_O_4_	C_10_H_16_O_4_	Betônica
5.8	40.81	165.0557		C_9_H_9_O_3_	C_9_H_10_O_3_	Coffee
5.9	86.04	195.0663		C_10_H_11_O_4_	C_10_H_12_O_4_	Aroeira
	37.29					Cipó-uva
5.9	42.46	279.1240		C_15_H_19_O_5_	C_15_H_20_O_5_	Velame
		363.1662		C_16_H_27_O_9_	C_16_H_28_O_9_	
6.1	48.13	401.1611 187.0975	179.0351	C_23_H_21_N_4_O_3,_	C_23_H_22_N_4_O_3_	Cipó-uva
		239.0926		C_9_H_15_O_4_,	C_9_H_16_O_4_	
		267.1241 279.1239		C_12_H_15_O_5,_	C_12_H_16_O_5_	
		345.1559		C_15_H_15_N_4_O,	C_15_H_16_N_4_O	
				C_15_H_19_O_5_,	C_15_H_20_O_5_	
				C_16_H_25_O_8_	C_16_H_26_O_8_	
6.6	100.00	199.0976		C_10_H_15_O_4_	C_10_H_16_O_4_	Betônica
	100.00					Pequi
	100.00					Cipó-uva
	74.76					Coffe
	75.88					Velame
	50.27					Aroeira
6.6	100.00	263.1290		C_15_H_19_O_4_	C_15_H_20_O_4_	Polifloral
6.8	44.47	201.1133		C_10_H_17_O_4_	C_10_H_18_O_4_	Betônica
	41.21					Pequi
	38.11					Cipó-uva
6.9	100.00	263.1292		C_15_H_19_O_4_	C_15_H_20_O_4_	Velame
	100.00					Aroeira
	100.00					Coffee
	85.28					Pequi
	52.63					Betônica

^a^ chromatographic data, ^b^ spectrometric data, ^c^ SmartFormula suggestion. RT—retention time, RA—relative area.

**Table 6:** Comparison with literature formulae for honeys.

m/z	MF	Suggested Compound	References	
315.051	C_16_H_12_O_7_	Rhamnetin	[32,33]	Betônica Velame Silvestre
299.056	C_16_H_12_O_6_	Kaempferide	[32,33]	Cipó-uva Coffee Velame
287.22	C_16_H_32_O_4_	Dihydroxypalmitic acid	[32]	Coffee Velame Silvestre
281.1394	C_15_H_22_O_4_	Syringic acid hexyl ester	[32]	Betônica Pequi Cipó-uva Coffee Velame Silvestre Aroeira
285.040	C_15_H_10_O_6_	Luteolin or Kaempferol	[32,34,35]	Betônica Coffee Cipó-uva Pequi Velame Silvestre
271.061	C_15_H_12_O_5_	Pinobanksin	[32,36]	Betônica Coffee Pequi Velame Silvestre
271.061	C_15_H_12_O_5_	Naringenin	[34,35]	Betônica Cipó-uva Pequi Velame Silvestre
269.045	C_15_H_10_O_5_	Galangin	[32,33]	Betônica Coffee Velame Silvestre
269.045	C_15_H_9_O_5_	Apigenin	[32,33]	Velame
263.129	C_15_H_20_O_4_	Abscisic acid	[32,33,34,36]	Betônica Aroeira Cipó-uva Pequi Velame Silvestre
255.066	C_15_H_12_O_4_	Pinocembrin	[32,33]	Velame Silvestre
253.050	C_15_H_10_O_4_	Chrysin	[32,33,34]	Betônica Aroeira Cipó-uva Velame Silvestre
201.113	C_10_H_18_O_4_	Dihydroxydecenoic acid	[32]	Betônica Cipó-uva Pequi Velame Silvestre
199.097	C_10_H_15_O_4_	Succinic acid	[32]	Betônica Pequi Aroeira Cipó-uva Coffee Velame Silvestre
195.066	C_10_H_12_O_4_	Hydroxyconiferyl alcohol	[32]	Aroeira Cipó-uva Silvestre
187.097	C_9_H_16_O_4_	Azelaic acid	[32]	Betônica Aroeira Coffee Cipó-uva Pequi Velame Silvestre
185.118	C_10_H_18_O_3_	Royal jelly acid	[32]	Betônica Pequi Aroeira Coffee
181.050	C_9_H_10_O_4_	Syringaldehyd	[32]	Betônica Coffee Aroeira Cipó-uva Pequi Velame Silvestre
179.035	C_9_H_8_O_4_	Cafeic acid	[32,33,34,35]	Betônica Aroeira Coffee Cipó-uva Pequi Velame Silvestre
169.014	C_7_H_6_O_5_	Gallic acid	[33,34]	Betônica Coffee Aroeira Cipó-uva Pequi Velame
165.055	C_9_H_10_O_3_	Phenyllactic acid	[32]	Betônica Aroeira Cipó-uva Coffee Pequi Velame Silvestre
163.040	C_9_H_8_O_3_	Coumaric acid	[33,34]	Coffee Aroeira Cipó-uva Pequi
151.040	C_8_H_8_O_3_	Vanillic acid	[35]	Betônica Coffee Aroeira Cipó-uva Pequi Velame Silvestre
144.045	C_9_H_7_NO	Quinolinol	[32]	Betônica Pequi Aroeira Cipó-uva Velame Silvestre
137.024	C_7_H_6_O_3_	p-hydroxybenzoic acid	[32]	Cipó-uva Coffee Velame Silvestre

**Table 7:** Data on the contents of total polyphenols and EC_50_ (mean ± SD; *n* = 3).

	mgGAE 100 g^−1^ of Sample	
	Total Polyphenols	EC_50_ (mg mL^−1^)
Betônica	57.62 ± 0.07 ^d^	76.21 ± 3.29 ^cd^
Pequi	54.37 ± 0.03 ^e^	105.56 ± 2.94 ^b^
Aroeira	74.74 ± 0.12 ^b^	68.81 ± 2.36 ^d^
Cipó-uva	40.70 ± 0.03 ^g^	150.71 ± 2.56 ^a^
Velame	70.06 ± 0.03 ^c^	51.48 ± 1.48 ^e^
Coffee	84.77 ± 0.05 ^a^	77.69 ± 3.55 ^c^
Polyfloral	52.37 ± 0.03 ^f^	72.84 ± 0.27 ^cd^

EC_50_ = 2.15 ± 0.01 μg mL^−1^ of gallic acid. Means followed by the same letter in the column do not differ according to Tukey’s test at *p* < 0.001.

**Table 8:** Results of minimum inhibitory concentration/minimum bactericidal concentration MIC/MBC.

Results (%) MIC/MBC							
	Cariogenic Bacteria						
	*E. faecalis*	*S. salivarius*	*S. sanguinis*	*S. sobrinus*	*S. mitis*	*L. paracasei*	*S. mutans*
Betônica	>20/>20	>20/>20	10/20	>20/>20	>20/>20	>20/>20	>20/>20
Pequi	>20/>20	20/20	20/20	20/>20	>20/>20	>20/>20	>20/>20
Aroeira	>20/>20	20/>20	10/20	>20/>20	>20/>20	>20/>20	>20/>20
Cipó-uva	>20/>20	>20/>20	10/>20	>20/>20	>20/>20	>20/>20	>20/>20
Velame	>20/>20	>20/>20	20/20	>20/>20	>20/>20	>20/>20	>20/>20
Coffee	>20/>20	>20/>20	20/>20	>20/>20	>20/>20	>20/>20	>20/>20
Polyfloral	20/20	10/10	10/10	10/10	20/20	10/10	10/10
Chlorhexidine	0.00074/ 0.00074	0.000092/ 0.000092	0.000092/ 0.000092	0.000046/ 0.000046	0.00018/ 0.00018	0.000092/0.000092	0.000046/ 0.000046

Concentrations of samples evaluated against cariogenic bacteria = 0.009% to 20%.

**Table 9:** Data for sample extraction and preparation.

	Grams	Mg Organic Phase	µL of Methanol/Water (3:2, *v*/*v*) *
Betônica	4.7	3.8	760
Pequi	4.9	3.4	680
Aroeira	4.9	5.0	1000
Cipó-uva	4.8	3.5	700
Coffee	4.8	4.8	960
Velame	4.7	3.3	660
Polyfloral	5.0	6.8	1360

* 5 µg/µL final concentration for all samples.

**Table 10:** Data for preparation of standards.

Standards	MF	MM	Total mg	µL of Methanol/Water (3:2, *v*/*v*)
gallic acid	C_7_H_6_O_5_	17,012	42.4	1030 µL
caffeine	C_8_H_10_N_4_O_2_	19,419	49.8	1030 µL
quercetin	C_15_H_10_O_7_	302,236	26.6	1026 µL
rutin	C_27_H_30_O_16_	610,517	17.2	1065 µL

MF—Molecular formula; MM—Molecular mass.

## Data Availability

Not applicable.

## Supplementary Materials

The following supporting information can be downloaded at: https://www.mdpi.com/article/10.3390/antibiotics11101429/s1, Figure S1: Boxplot pattern of honeys in relation to total polyphenols analysis and antioxidant activity, Figure S2: UHPLC chromatographic profile of Betônica honey, Figure S3. UHPLC chromatographic profile of Pequi honey, Figure S4: UHPLC chromatographic profile of Aroeira honey, Figure S5: UHPLC chromatographic profile of Cipó-uva honey, Figure S6: UHPLC chromatographic profile of Coffee honey, Figure S7: UHPLC chromatographic profile of Velame honey, Figure S8: UHPLC chromatographic profile of Plyfloral honey.
