# Supplementary material for: Physicochemical Profile, Antioxidant and Antimicrobial Activities of Honeys Produced in Minas Gerais (Brazil)
Source: Antibiotics (Basel). 2022 Oct 18;11(10):1429. doi: 10.3390/antibiotics11101429 (PMC9598309; doi:10.3390/antibiotics11101429)
Supplement: Supplementary file 1 [file antibiotics-11-01429-s001.zip › antibiotics-1975595-supplementary-final.pdf]

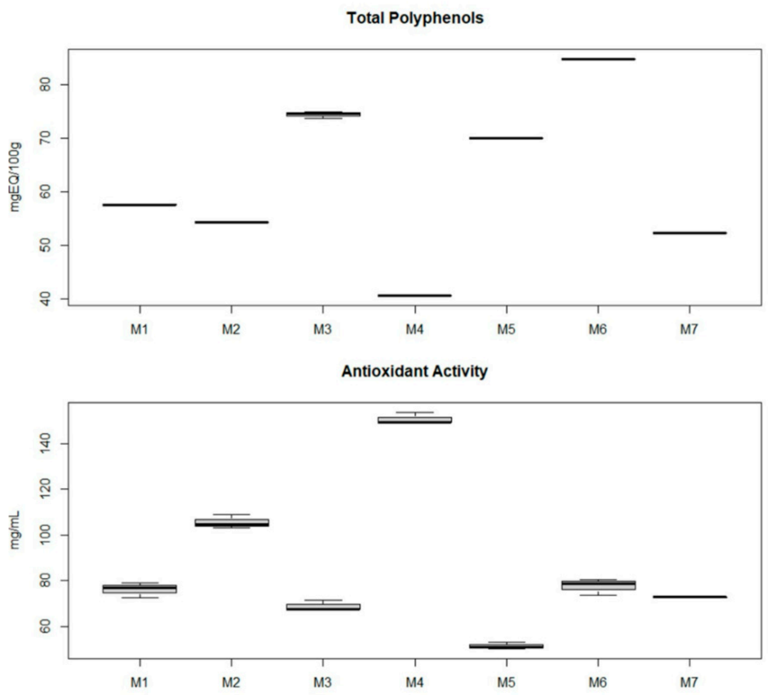

Figure S1. Boxplot pattern of honeys in relation to total polyphenols analysis and antioxidant activity.

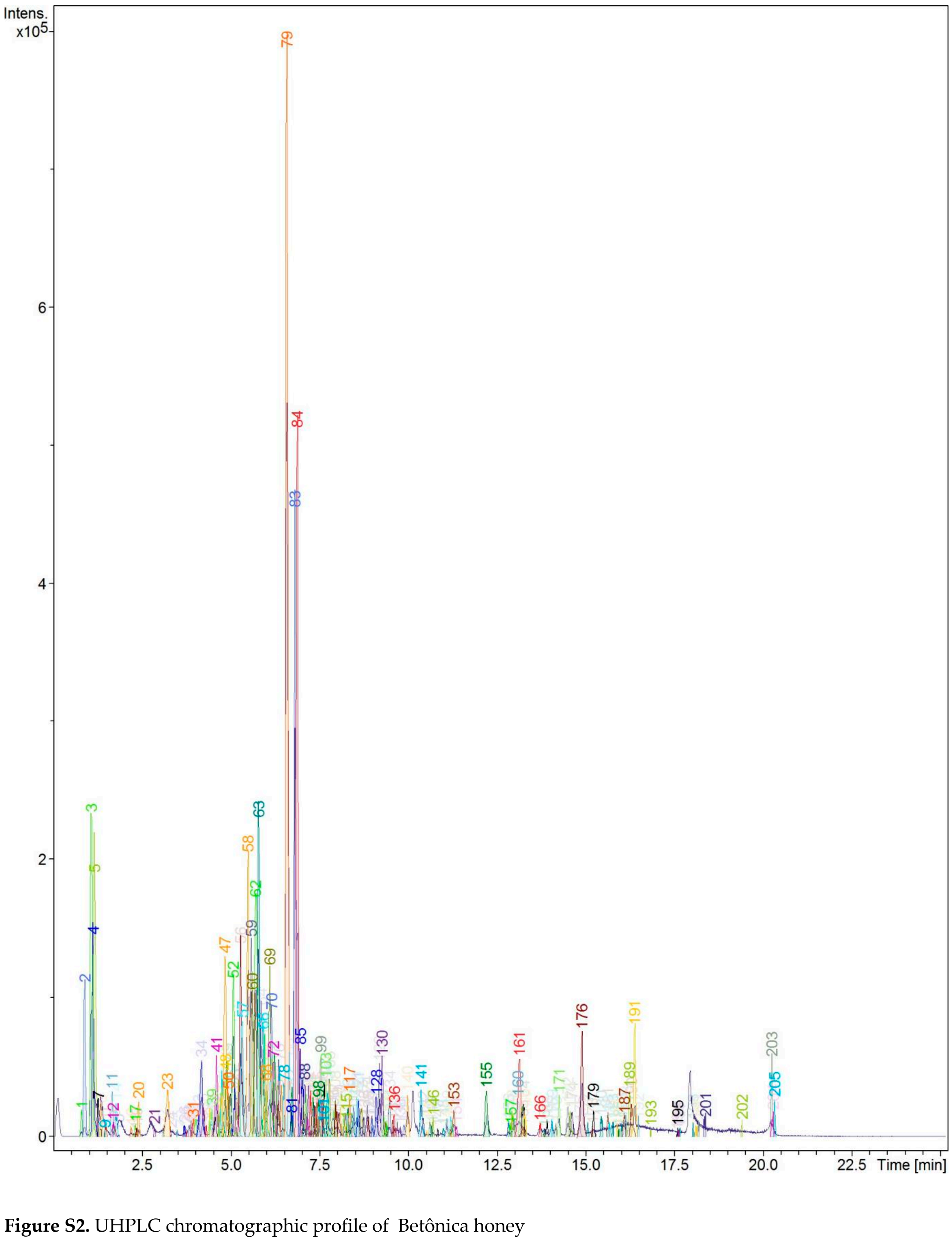

Figure S2. UHPLC chromatographic profile of Betônica honey

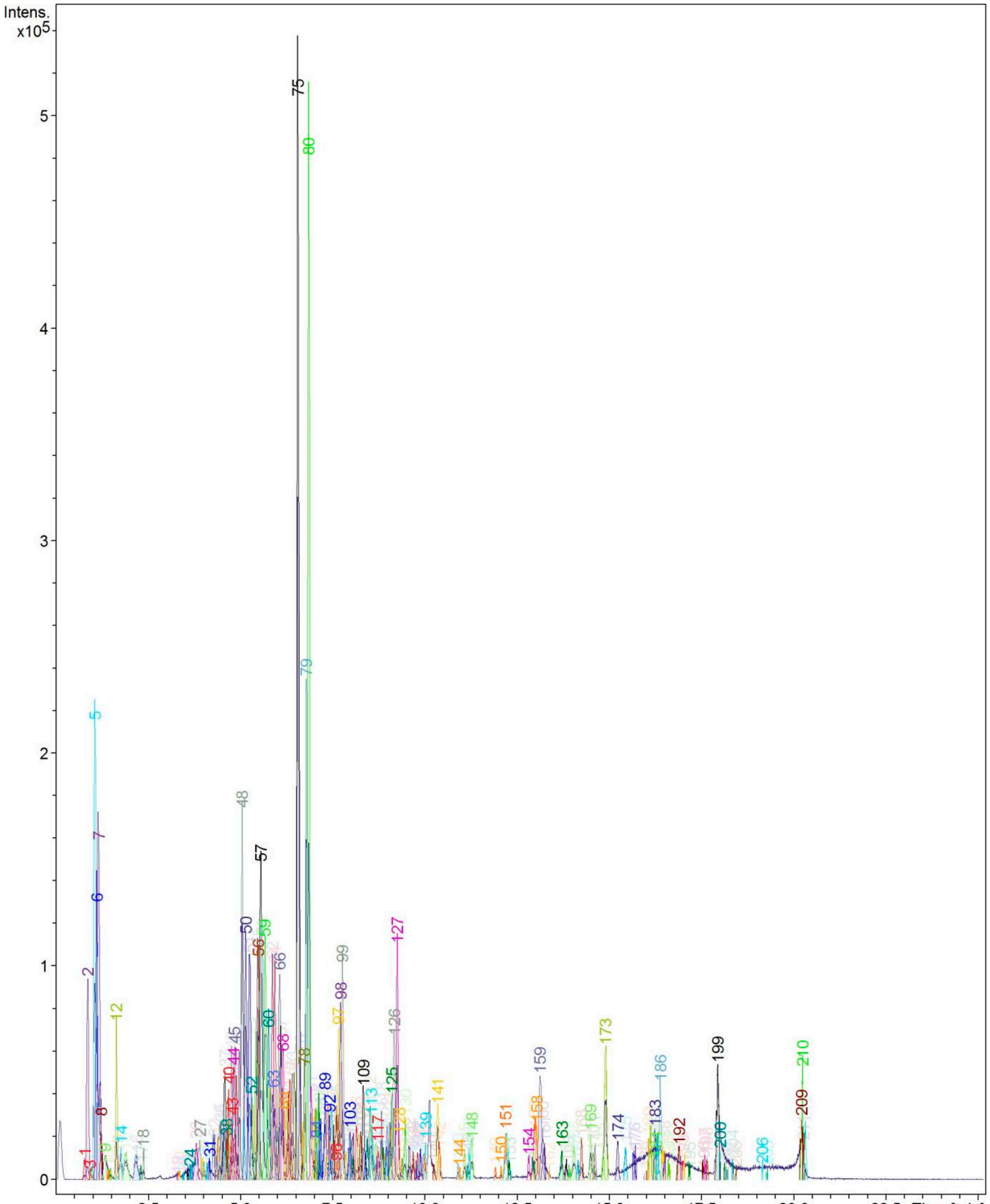

Figure S3. UHPLC chromatographic profile of Pequi honey.

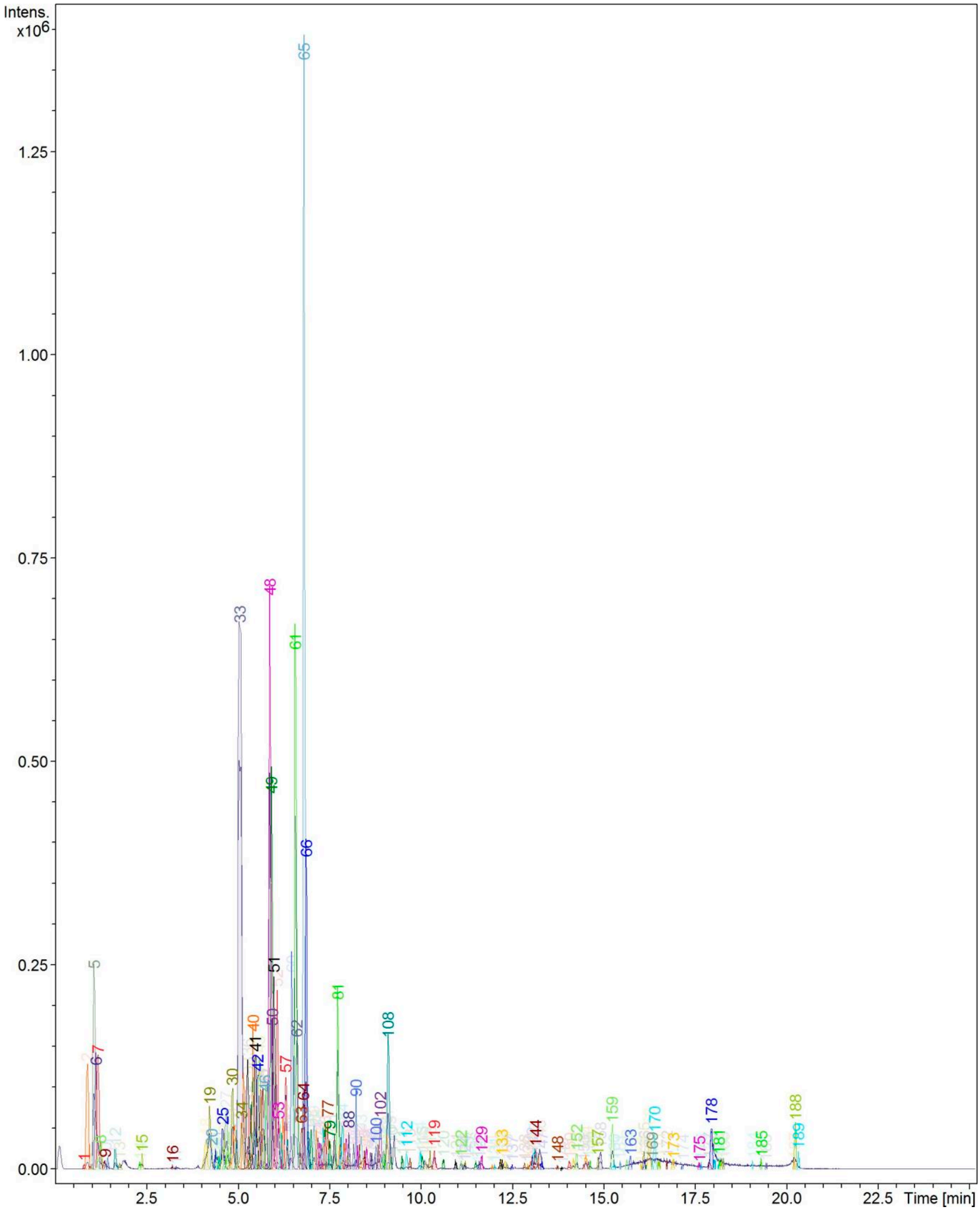

Figure S4. UHPLC chromatographic profile of Aroeira honey.

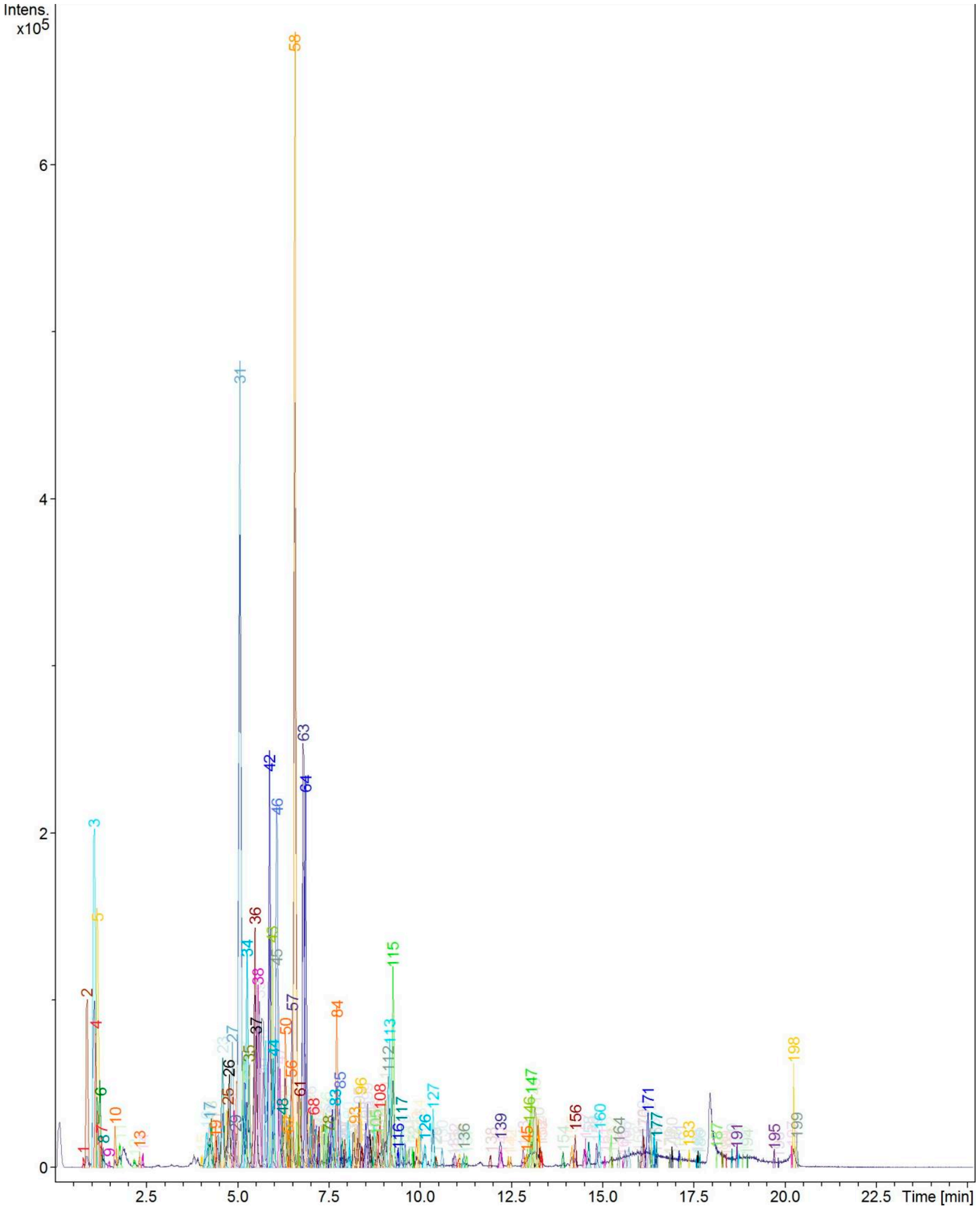

Figure S5. UHPLC chromatographic profile of Cipó-uva honey.

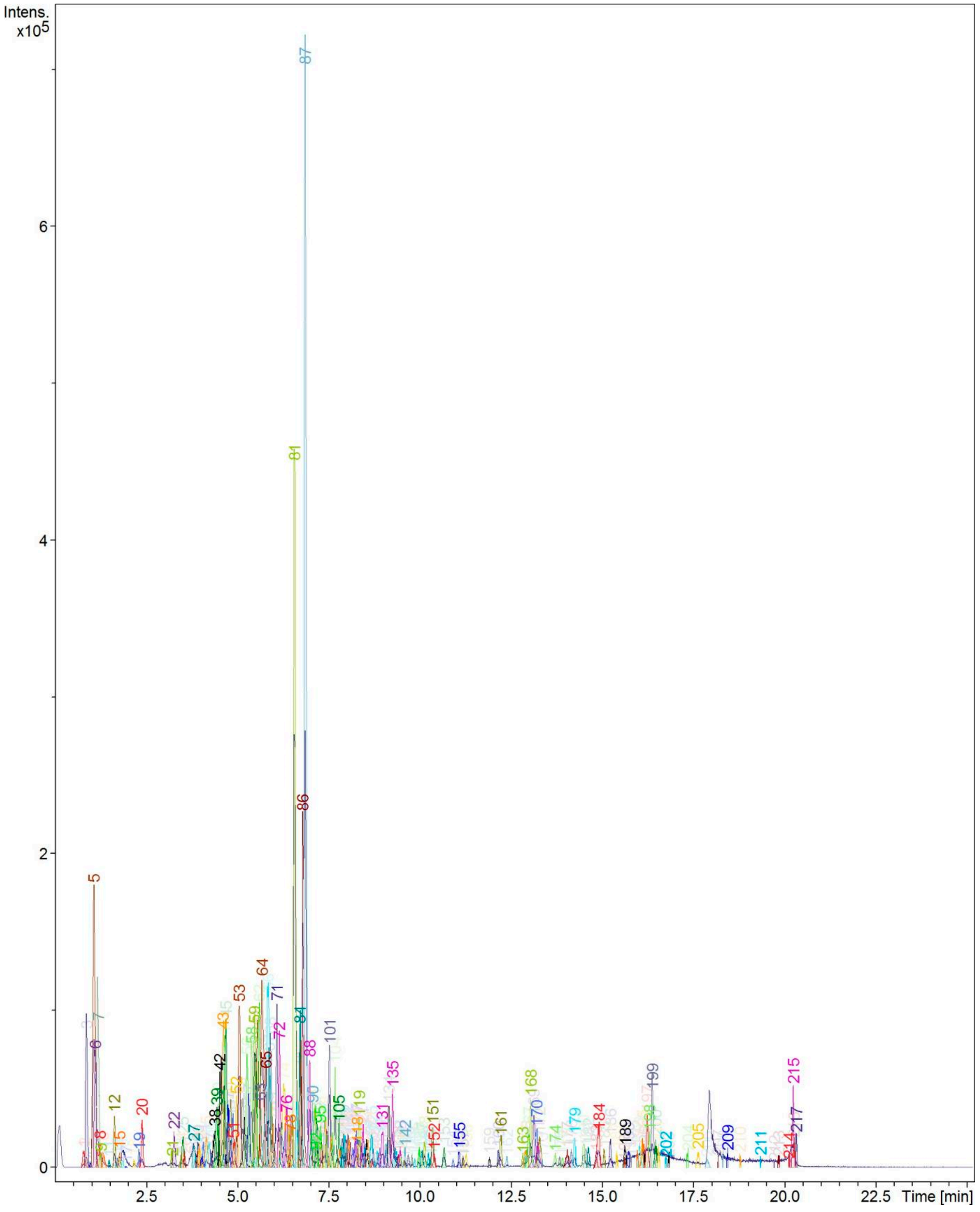

Figure S6. UHPLC chromatographic profile of Coffee honey.

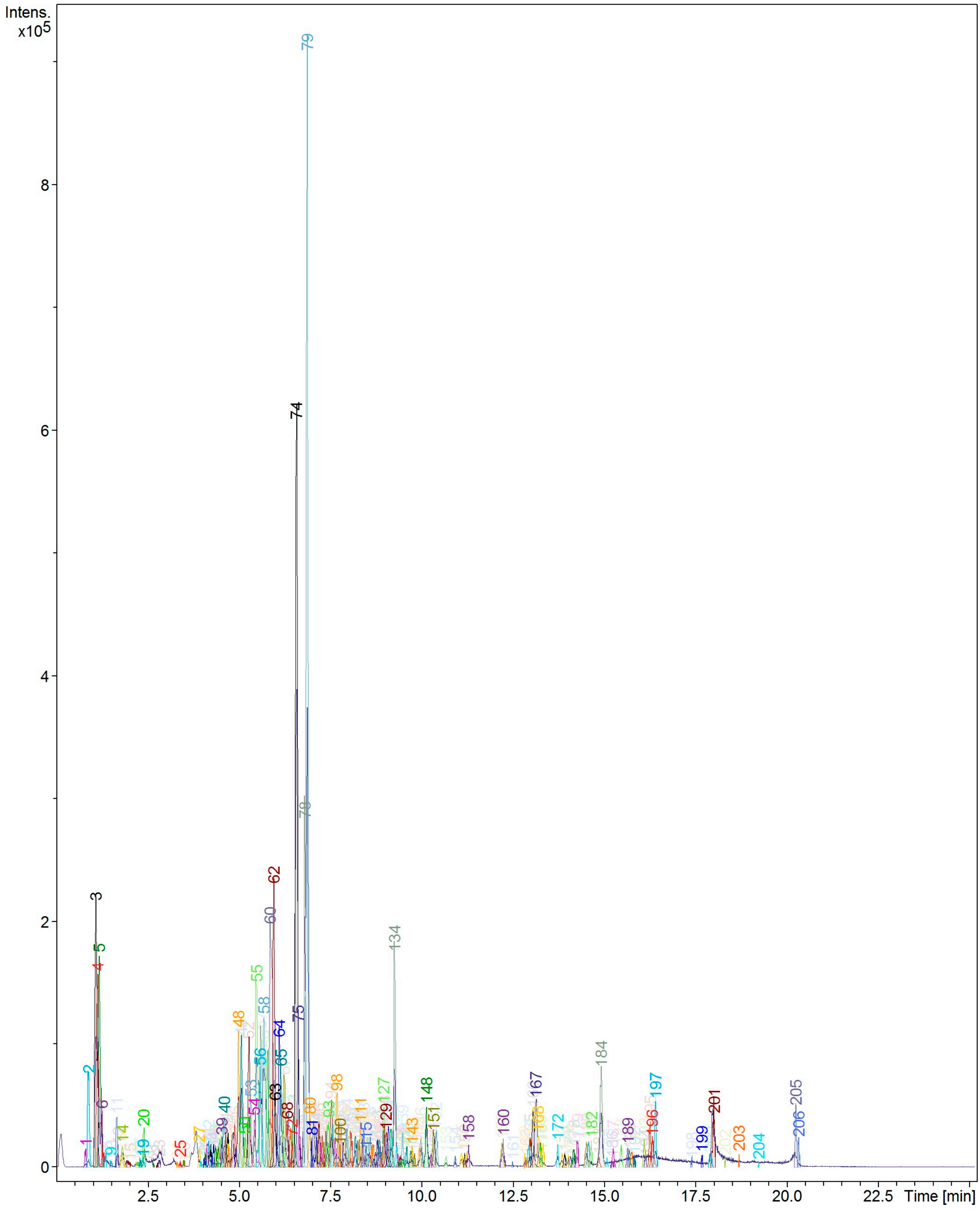

Figure S7. UHPLC chromatographic profile of Velame honey.

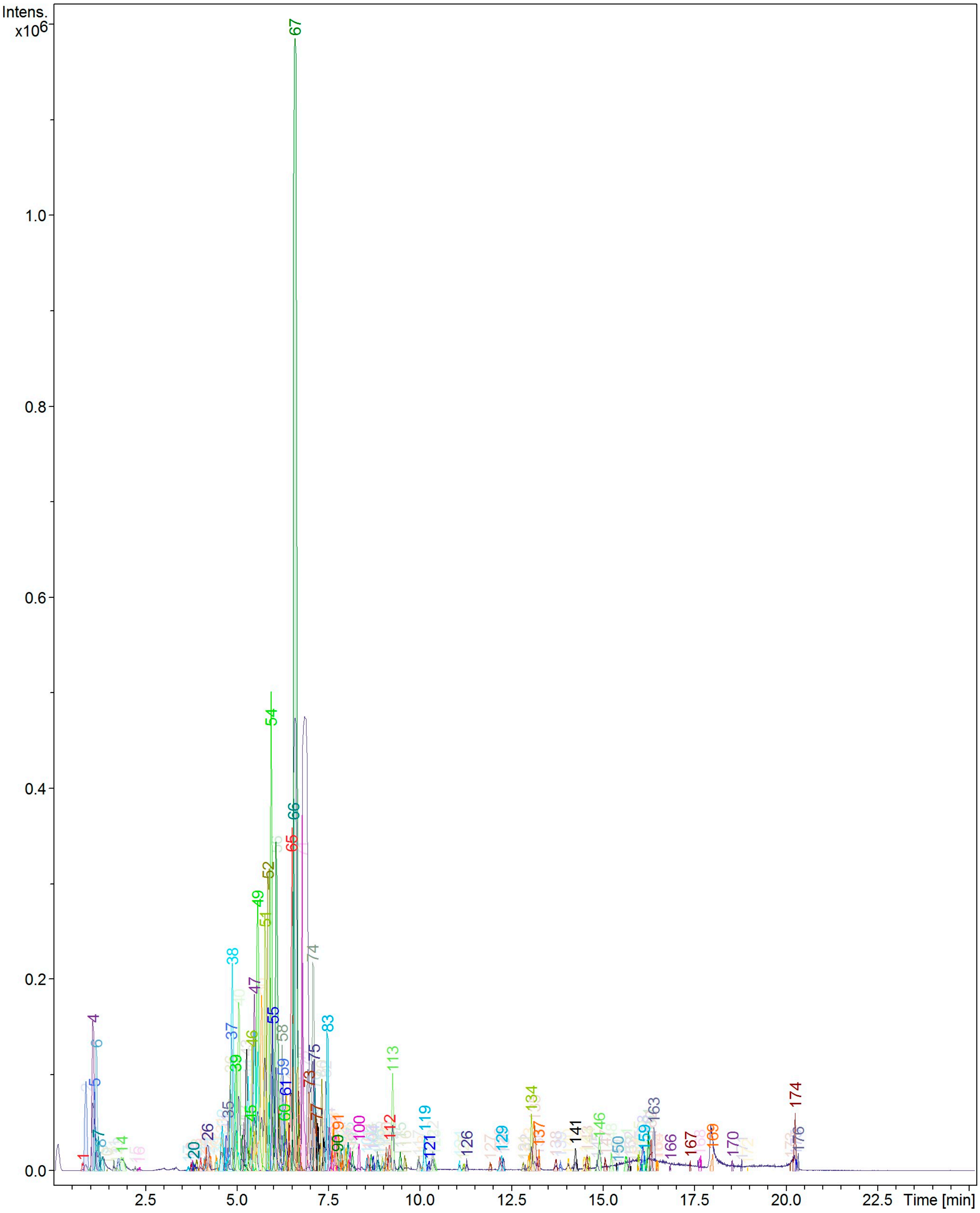

Figure S8. UHPLC chromatographic profile of Plyfloral honey.
